# Small-Scale Soil Microbial Community Heterogeneity Linked to Landform Historical Events on King George Island, Maritime Antarctica

**DOI:** 10.3389/fmicb.2018.03065

**Published:** 2018-12-10

**Authors:** Yumin Zhang, Lu Lu, Xulu Chang, Fan Jiang, Xiangdong Gao, Yifeng Yao, Chengsen Li, Shunan Cao, Qiming Zhou, Fang Peng

**Affiliations:** ^1^China Center for Type Culture Collection (CCTCC), College of Life Sciences, Wuhan University, Wuhan, China; ^2^College of Life Sciences, Wuhan University, Wuhan, China; ^3^State Key Laboratory of Systematic and Evolutionary Botany, Institute of Botany, Chinese Academy of Sciences, Beijing, China; ^4^SOA Key Laboratory for Polar Science, Polar Research Institute of China, Shanghai, China; ^5^ChosenMed Technology (Beijing) Company Limited, Jinghai Industrial Park, Economic and Technological Development Area, Beijing, China

**Keywords:** soil-borne microbial community, small-scale spatial heterogeneity, landform, maritime Antarctica, Miseq sequencing platform, phospholipid fatty acid

## Abstract

Although research on microbial biogeography has made great progress in the past decade, distributions of terrestrial microbial communities in extreme environments such as Antarctica are not well understood. In addition, knowledge of whether and how historical contingencies affect microbial distributions at small spatial scales is lacking. Here, we analyzed soil-borne microbial (bacterial, archaeal, and fungal) communities in 12 quadrat plots around the Fildes Region of King George Island, maritime Antarctica, and the communities were divided into two groups according to the soil elemental compositions and environmental attributes of Holocene raised beach and Tertiary volcanic stratigraphy. Prokaryotic communities of the two groups were well separated; the prokaryotic data were primarily correlated with soil elemental compositions and were secondly correlated with environmental attributes (e.g., soil pH, total organic carbon, NO_3_^-^, and vegetation coverage; Pearson test, *r* = 0.59 vs. 0.52, both *P* < 0.01). The relatively high abundance of P, S, Cl, and Br in Group 1 (Holocene raised beach site) was likely due to landform uplift. Lithophile-elements (Si, Al, Ca, Sr, Ti, V, and Fe) correlated with prokaryotic communities in Group 2 may have originated from weathering of Tertiary volcanic rock. No significant correlations were found between the fungal community distribution and both the soil elemental composition and environmental attributes in this study; however, Monte Carlo tests revealed that elements Sr and Ti, soil pH, sampling altitude, and moss and lichen species numbers had significant impacts on fungal communities. The elements and nutrients accumulated during the formation of different landforms influenced the development of soils, plant growth, and microbial communities, and this resulted in small-scale spatially heterogeneous biological distributions. These findings provide new evidence that geological evolutionary processes in the Fildes Region were crucial to its microbial community development, and the results highlight that microbial distribution patterns are the legacies of historical events at this small spatial scale. Based on this study, the ice-free regions in maritime Antarctica represent suitable research sites for studying the influence of geomorphological features on microbial distributions, and we envision the possibility of a site-specific landform assignment through the analysis of the soil prokaryotic community structure.

## Introduction

Investigations of microbial communities at different spatial scales and the factors that affect their distributions are fundamental aspects of microbial biogeography (Martiny et al., 2006; Lozupone and Knight, 2007). Up to now, a growing body of research has revealed that microorganisms are not randomly distributed and are influenced by two types of deterministic factors—contemporary environmental attributes and historical contingencies—a classic theory that can be used to explain the diversity and distribution of microorganisms (Martiny et al., 2006).

Numerous studies have shown that microbial assemblages can be affected by various environmental disturbances (Comte et al., 2016). In many terrestrial ecosystems, bacterial, fungal, and archaeal communities are distributed along soil parameter gradients (e.g., temperature, pH, water content, salinity, and nutrient concentrations) (Walsh et al., 2005; Yergeau et al., 2007a; Lauber et al., 2009; Rousk et al., 2010; Lin et al., 2012). Meanwhile, plants and animals that depend on the soil ecosystem may also have significant influences on microorganisms (Zhang and Blackwell, 2002; Falush et al., 2003; Teixeira et al., 2010). Therefore, microbial communities can be influenced by numerous interdependent abiotic and biotic factors. In most distinct areas with special environments, such as acidic aqueous and saline environments, distribution trends in the microbial community are shaped dominantly by environmental factors that limit or prevent cell growth (Lozupone and Knight, 2007; Kuang et al., 2013). Previous studies suggest that habitat specialization plays a pivotal role in the microbial community composition, and this is related to deterministic processes driven by contemporary environmental heterogeneity (Wang et al., 2013). Historical contingencies, in which the biotic composition reflects the legacies of historical events, are other types of deterministic factors that are important for the microbial community distribution at large spatial scales (one to tens of thousands of kilometers) (Martiny et al., 2006). However, in contrast to the increasing amount of research on other topics related to microbial distributions, knowledge about historical contingency effects is lacking (Fukami, 2015), and in particular, historical contingency effects on microbial community distributions at small spatial scales have not been elucidated thoroughly yet. This lack of knowledge may be due in part to the large spatial scales of influence of one historical event, i.e., there may be little variation over a small spatial region, and moreover, the tendency for legacy effects of a historical event to be overwhelmed by any effect from contemporary environmental factors at small spatial scales.

The extreme conditions of Antarctica, such as low temperatures, low nutrient availability, high UV radiation, and frequent freeze–thaw activity (Wynnwilliams, 1990; Convey, 1996), result in relatively simple ecosystems. Hence, the relatively uncomplicated food-web structure of Antarctic terrestrial habitats provides an appropriately manageable system to investigate the drivers of soil microbial diversity and composition (Yergeau et al., 2009). Unsurprisingly, spatial microbial community patterns have been observed here, and these range from patterns at site-specific regions to those at large regional scales (Webb et al., 2002; Yergeau et al., 2007b; Chong et al., 2010). The Fildes Region, King George Island, is one of the largest ice-free regions in maritime Antarctica, and it has a higher biodiversity than continental Antarctica. This typical small-scale spatial region includes two Antarctic Special Protected Areas (ASPAs) covering approximately 30 km^2^ of the Fildes Peninsula, Ardley Island, and adjacent islands (Braun et al., 2012). After the Last Glacial Maximum (LGM), this region experienced multiple geologic and glacial events including deglaciation (8400–5500 BP), glacial re-advance (after 6000 BP), Holocene glacio-isostatic and tectonic uplift during the glacial erosive phase, and glacier retraction (Ingolfsson et al., 1998; Michel et al., 2014). Glacial activities and past sea level changes were the key drivers of landform and soil development across the Fildes Region (Boelter, 2011; Watcham et al., 2011), and the effects of these historical events on terrestrial microbial communities should not be ignored (Viles, 2012).

In this study, we used the Illumina Miseq sequencing platform and the phospholipid fatty acids (PLFA) method to survey the diversity and structure of prokaryotic and fungal communities in 12 quadrat plots around the Fildes Region, King George Island. Meanwhile, we attempted to identify the deterministic factors that have driven the microbial distribution. The 12 permanent quadrats analyzed in our study have been established since 2013. The primary aim of constructing these plots was to establish a site for long-term evaluations of ecosystem evolution via biomass and diversity indicators under climate change conditions and to build a comprehensive research platform for multi-disciplinary research including botany, microbiology, ecology, and environmental science (Yao et al., 2017). For these purposes, all of the selected quadrats have the following characteristics: (a) they must include Antarctic hairgrass (*Deschampsia antarctica*), the only advanced plant discovered in the Fildes Region, which is associated with moss and lichen; (b) they must have stable soil and vegetation for long term monitoring; and (c) they must be protected from human disturbance (mostly scientific explorers) and animal activity as much as possible. Soil maturation and vegetation colonization takes a very long time under unfavorable conditions, so the established quadrats represented natural and stable habitats around the region. Recently, based on 454 pyrosequencing data, Wang et al. (2015) found that the diversity and structure of soil bacterial communities in four sites of the Fildes Region were affected significantly by the pH, phosphate phosphorus, organic carbon, and organic nitrogen. However, the relationships among microbial communities, geological factors, and landform development have not been studied. In this study, we attempt to answer the following three questions: (i) what is the microbial community structure in this small-scale region of maritime Antarctica; (ii) do the microbial communities display heterogeneity here; and (iii) do the microbial community distributions relate to landform historical contingencies in the Fildes Region. In order to explore these issues, a number of soil chemical properties and vegetation attributes were measured, which represent conventional environmental factors; additionally, the soil elemental composition was determined by X-ray fluorescence spectrometry, as such data are deemed to be an acceptable proxy for soil or sediment erosion and development in different landforms (Xie and Ren, 1993; Battaglia et al., 2003; Guilizzoni et al., 2006), and we combined our results with the geological literature related to the Fildes Region to represent the landform types. Our aim was to improve the understanding of terrestrial microbial communities in maritime Antarctic ice-free areas and to contribute a new perspective on small-scale microbial biogeography.

## Materials and Methods

### Quadrat Plot Description, Soil Sampling, and Sample Preparation

The Fildes Region is the largest ice-free area on King George Island, and it has a humid and relatively mild sub-Antarctic maritime climate. The mean annual temperature and precipitation are -2.4°C and over 500 mm, respectively (Gerighausen et al., 2003). The 12 permanent quadrat plots (1.5 m × 1.0 m each) investigated in this study were established on the Fildes Peninsula and Ardley Island between 2013 and 2015. Each quadrat plot was fenced to minimize disturbance. GPS coordinates, vegetation characteristics, and the landscape of quadrat locations are shown in Table 1 and Supplementary Figure S7. The distance between quadrat plots ranges from approximately 1.6 to 8.2 km. Sampling occurred during China’s 33rd Antarctic expedition in January 2017. Soils were sampled from the A-horizon (10 cm) at an internal distance of approximately 3–5 m, and samples were collected in triplicate around each quadrat plot. Soil samples collected for each replicate were taken from five soil cores (5 cm diameter) and mixed thoroughly. A total of 36 soil samples were placed in sterile plastic bags, and soil DNA was extracted within 2 h in the laboratory of the Great Wall Station. The remaining soils were stored in the freezer until further soil physico-chemical property analyses were performed.

**Table 1:** Locations and partial vegetation properties of the 12 soil quadrats.

Quadrate code	Coordinates	Elevation/(m.a.s.l)	Aspect^∗^	Number of grass tufts^∗^	Grass cover/%^∗^	Moss cover/%^∗^	Lichen cover/%^∗^
Q1	62°12′39″S 59°00′49″W	11	NW	26	20.75	55	10
Q2	62°12′39.6″S 58°55′35.9″W	34	N	30	11	46	43
Q3	62°11′05.1″S 58°52′37.3″W	22	NE	>50	37.50	56	6
Q4	62°12′00″S 58°59′40″W	42	NW	46	20	15	7
Q5	62°10′13″S 58°55′26″W	50	NW	1	1.75	35	5
Q6	62°13′00″S 58°57′52″W	42	NE	4	14	40	45
Q7	62°11′00.4″S 58°51′28.6″W	47	NE	>100	50	40	10
Q9	62°11′20″S 58°55′10″W	42	NW	24	10	20	10
Q10	62°09′09.1″S 58°55′44.2″W	37	NW	17	31	60	1
Q11	62°09′57.4″S 58°57′59.4″W	32	NW	2	1.50	–	20
Q12	62°10′33″S 58°58′16″W	43	NW	1	1.50	10	30
Q13	62°11′45.6″S 58°56′21.1″W	56	NE	1	2.50	5	25


*^∗^Data are from the previous study (Yao et al., 2017).*

### DNA Extraction, PCR, Illumina Miseq Sequencing, and Sequencing Data Treatment

Genomic DNA was extracted by using a PowerSoil DNA Isolation Kit (Mo Bio, Carlsbad, CA, United States) according to the manufacturer’s instructions. Duplicate DNA extraction was performed for each sampling plot, and all duplicated DNA products were pooled to reduce potential DNA extraction bias. Afterwards, DNA concentrations were measured with a UV spectrophotometer (Eppendorf, Bio Photometer) and molecular sizes of the DNA were estimated by 0.8% agarose gel electrophoresis. Details of the Illumina Miseq sequencing and sequencing data treatment are described in Appendix S1. These sequence data have been submitted to the DDBJ/EMBL/GenBank databases (SRA) under accession no. SRP132288, accession no. SRP132345, and accession no. SRP132350.

### PLFA Analysis

Phospholipid fatty acids (PLFAs) from soil samples were extracted, fractionated, quantified, and analyzed by using the protocols described in an earlier study (Bossio and Scow, 1995). In brief, 2.0 g of soil (dry weight) was extracted with a chloroform–methanol–citrate buffer mixture (1:2:0.8) and fractionated into neutral lipids, glycolipids, and phospholipids on a silicic acid column (Agilent Technologies, Sillic Box, CA, United States). Phospholipids were subjected to mild alkaline methanolysis after separating out fatty acid methyl esters on an Agilent 6890N gas chromatograph equipped with a flame ionization detector and an HP-1 Ultra 2 capillary column (Agilent Technologies, Santa Clara, CA, United States). Peak areas were quantified by adding methyl non-adecanoate fatty acid (C19:0) (Sigma) as an internal standard. The fatty acid methyl esters were prepared according to the MIDI protocol and analyzed by using the MIDI Sherlock Microbial Identification System (MIDI, Newark, DE, United States). The fatty acids *i*14:0, *i*15:0, *a*15:0, *i*16:0, *a*16:0, *i*17:0, and *a*17:0 represented Gram-positive bacteria, whereas 16:1*ω*9*c*, *cy*17:0, 18:1*ω*5*c*, 18:1*ω*7*c*, and *cy*19:0 represented Gram-negative bacteria; furthermore, 10Me16:0 (Frostegard and Baath, 1996), 10Me17:0, and 10Me18:0 represented Actinomycete (Zelles, 1997), branched monoenoic and mid-branched saturated fatty acids represented anaerobic microorganisms (Zhong et al., 2010), and 16:1*ω*5 represented arbuscular mycorrhiza (AM) fungi (Olsson et al., 1999). The PLFAs were categorized and calculated in the MIDI Sherlock Microbial Identification System (MIDI, Newark, DE, United States).

### Soil Elemental Composition Determined by X-Ray Fluorescence Spectrometry

Soil samples were dried at 105°C for 6 h and then ground into a powder. The soil powder was pressed in a 45 mm bore steel die under an approximately 20 t hydraulic press. Afterwards, every soil sample had formed into a stable soil pie 45 mm in diameter and 10 mm in height. These pies were generally analyzed within a few hours. The elements within soil samples were determined by X-ray fluorescence spectrometry (Bruker AXS, Germany) with a standardless quantitative analysis method (Handley et al., 2010). We removed poor quality elemental signals that appeared rarely in the data (<0.01%), and these amounted to only one or two samples.

### Soil Parameters and Vegetation Attribute Measurements

Soil temperature was measured by a plug-type thermometer (ZD Instrument, China) at depths of 15 cm during soil sampling. Soil pH was measured by adding 10 mL of distilled water to 5 g of soil and recording the pH with a pH electrode (Mettler-Toledo, Switzerland). Soil moisture was determined as the gravimetric weight loss after drying the soil at 105°C until it reached a constant weight. Analysis of total organic carbon (TOC) was performed by using a TOC analyzer (vario TOC, Elementar, Germany). To measure NH_4_^+^ and NO_3_^-^, 10 g of soil was suspended in 50 mL of a 2 mol/L KCl solution and shaken at 25°C for 1 h. Then, the soil solution mixture was centrifuged for 5 min at 3000 *g*. Subsequently, the clear supernatant was passed through a filter of 0.45 μm (Millipore, type GP) and analyzed by using a continuous flowing analyzer (FIAstar 5000, Foss, Denmark). Within each quadrat of 1 m × 1 m part, we measured the vegetation attributes including moss species number (MS), lichen species number (LS), hairgrass (*D. antarctica*) coverage (DAC), and total vegetation coverage (VC) according to previous protocols (Yao et al., 2017).

### Statistical Analyses

For estimating the bacterial, archaeal, and fungal diversity, operational taxonomic unit (OTU) analyses were carried out with the Shannon, Chao 1, and ACE indices; this was accomplished by using the Mothur v. 1.30.2 software package (Schloss et al., 2009). The relationships between soil elemental compositions and environmental attributes in the 32 soil samples were analyzed by principal component analysis and hierarchical clustering heatmap analysis with R v. 3.3.1 statistical software. The Wilcoxon test was performed on the soil elements and environmental data to determine the level of significance with a two-sided hypothesis by using the Statistical Package for the Social Sciences (SPSS) software. Significant differences in soil elemental compositions, environmental attributes, and microbial community structures between groups were determined by permutational multivariate analysis of variance (PERMANOVA) on 999 permutations of residuals under a reduced model by using R v. 3.3.1 statistical software. The Bray–Curtis distance was used to obtain the dissimilarity matrices in the PERMANOVA test for microbial OTU data, which had been normalized by dividing the reads per OTU in a sample by the sum of usable reads in that sample (relative abundances), where an OTU absent from a sample was coded as state 0. This normalized method was also used for processing PLFA data in significance tests and the canonical correspondence analysis (CCA). According to the detrended correspondence analysis (DCA, maximum of axis lengths > 3), CCA was the most appropriate method for the constrained ordinations obtained with all of the community data. The similarity test, Mantel test, and CCA were used to evaluate the linkages between microbial community structures (general levels) and soil elemental compositions and environmental attributes with the Vegan package (v. 2.4-1) in R v. 3.3.1 according to the methods described in earlier studies (Yang et al., 2014). Variation inflation factors were used to select factors in CCA modeling, of which the variance of canonical coefficients was not inflated by the presence of correlations with other factors, so that soil elements and environmental attributes were removed if the variation inflation factor was larger than 20. Variation partitioning analysis resulted in 11 soil elements (Si, Ca, Zn, Fe, Al, Mn, V, Ti, Sr, P, and Br) and 10 environmental attributes. The effects of factors on microbial community structures and the PLFA profiles were estimated by a Monte Carlo permutation test (999 permutations). Differences in microbial categories marked by PLFAs were determined by using Welch’s *t*-test (two-sided) with the STAMP software (v. 2.1.3) package.

## Results

### Soil Elemental Compositions and Environmental Attributes of Quadrats

**FIGURE 1:**
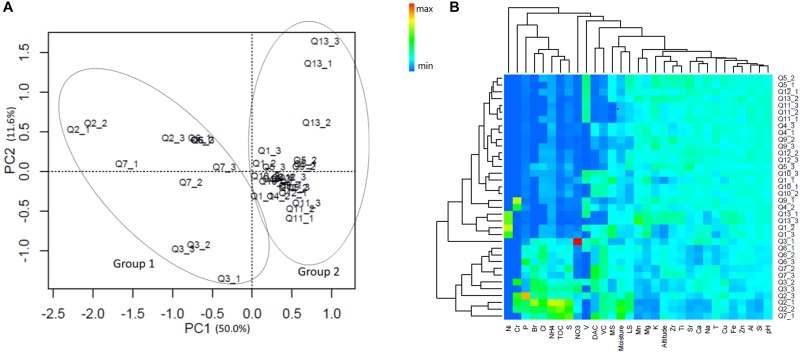
**(A)** Principle component analysis (PCA) and **(B)** heatmap cluster analysis of the normalized soil elemental compositions and environmental attribute data. The values of PC1 and 2 are percentages of total variations that can be attributed to the corresponding axis. T, temperature; TOC, total organic carbon; MS, moss species amount; LS, lichen species amount; DAC, hairgrass (*Deschampsia antarctica*) coverage; and VC, total vegetation coverage.

A total of 20 elements in the sample soils were detected by X-ray fluorescence spectrometry, and 11 environmental attributes were measured (Supplementary Table S1). Principle component analysis with normalized whole soil elemental compositions and environmental attribute data by their root mean square showed that the 36 samples were well separated by the original point of the PC1 coordinate axis (Figure 1A). Hence, the quadrat plots could be divided into two groups according to the different soil types and environmental conditions; Group 1 included quadrat plots Q2, Q3, Q6, and Q7, and Group 2 included all other quadrat plots (Q1, Q4, Q5, Q9, Q10, Q11, Q12, and Q13). The heatmap cluster analysis supported the grouping suggested by the whole element and environmental data analyses (Figure 1B).

The soil elemental profiles revealed that lithophile elements (Si, Al, Ca, Mg, and Fe) constituted the major portions. Twelve elements (Al, Ca, Cu, Fe, K, Mg, Mn, Si, Sr, Ti, V, and Zn) were significantly more abundant in Group 2, and four elements (P, S, Cl, and Br) were significantly more abundant in Group 1 (Wilcoxon test, *P* < 0.05, Supplementary Table S1). The PERMANOVA analysis revealed a highly significant difference in soil elemental composition between the two sample groups (*Pseudo-F* = 17.74, *P* < 0.01). Pairwise correlative comparisons between elements demonstrated that P, S, Cl, and Br were positively correlated with each other, and they were negatively correlated with Mg, Al, Si, Ca, Mn, Zn, Sr, V, Fe, and Ti, which also displayed positive correlations with each other (Supplementary Figure S1). Significant differences were also observed in the environmental attributes between Group 1 and Group 2 (PERMANOVA test, *Pseudo-F* = 15.17, *P* < 0.01), and these consisted of lower soil pH values and higher total organic carbon (TOC), NH_4_^+^, NO_3_^-^, and moisture contents in Group 1. In addition, vegetation properties such as DAC and VC were also higher in Group 1 plots (Wilcoxon test, *P* < 0.05, Supplementary Table S1).

### Diversity and Composition of the Microbial Communities in Quadrats

**FIGURE 2:**
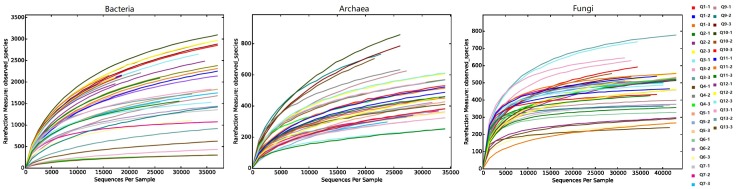
Rarefaction curves of phylotype richness (number of unique OTUs).

**FIGURE 3:**
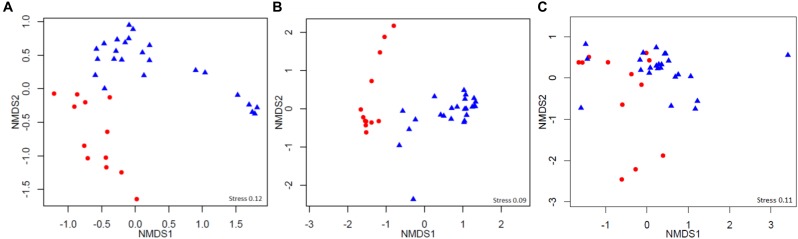
Non-metric multidimensional scaling (NMDS) (Bray–Curtis similarity) analysis of the **(A)** bacterial, **(B)** archaeal, and **(C)** fungal operational taxonomic unit (OTU) datasets. Circle = Group 1; triangle = Group 2.

After sequence-quality filtering, we obtained the following totals of rarefied reads: 2,389,662 high-quality bacterial 16S rRNA gene reads, 1,423,619 archaea 16S rRNA gene reads, and 1,953,908 internal transcribed spacer (ITS) reads. These reads constituted 98,887, 49,000, and 8,464 rarified OTUs at a 0.03 discrepancy (97% identity) for bacterial, archaeal, and fungal taxa, respectively. The OTU diversities of the Shannon, Chao 1, and ACE indices for bacteria, archaea, and fungi did not differ between Group 1 and Group 2 (Wilcoxon test, *P* > 0.05, Supplementary Table S2). Rarefaction curves of phylotype richness (number of unique OTUs) for all samples are presented in Figure 2. For bacteria, 20 phyla and some unidentified bacteria (0–0.8%) were detected, and the OTU sequences of most quadrat soils were dominated by Actinobacteria (24.2%), Acidobacteria (14.7%), Proteobacteria (15.1%), Chloroflexi (12.3%), and Gemmatimonadetes (7.2%) (Supplementary Figure S2). For archaea, a number of OTU sequences (74.6%, maximum) were not assigned to any taxon; the remainder was dominated by Crenarchaeota (94.5%) and Euryarchaeota (5.5%), and the dominant classes of the phyla were Thaumarchaeota and Thermoplasmata, respectively. For fungi, six phyla were detected, and all quadrat plot soils were dominated by Ascomycota (69.1%), Basidiomycota (17.6%), and Zygomycota (4.5%). The percentage of unassigned OTUs and unidentified fungi were 6.8–64.3% and 0.4–7.7%, respectively. The appearance of unidentified fungi may have been due to the limited lengths of the ITS reads and the lack of reference sequences from well-identified fungi in the database, such as some mycorrhizal fungi (Ryberg et al., 2008; Nilsson et al., 2009). PERMANOVA tests of Bray–Curtis distances revealed that there were significant differences between the two groups for prokaryotic 16S rRNA genes (integrated data normalized by the bacterial and archaeal OTU datasets, *Pseudo-F* = 3.1, *P* = 0.0002), but not for fungal ITS genes (*Pseudo-F* = 1.3, *P* = 0.196). This was consistent with the grouping results of the non-metric multidimensional scaling (NMDS) analysis with the bacterial and archaeal OTU datasets (Figure 3).

### Links Among Microbial Composition, Soil Elemental Composition, and Environmental Attributes

**FIGURE 4:**
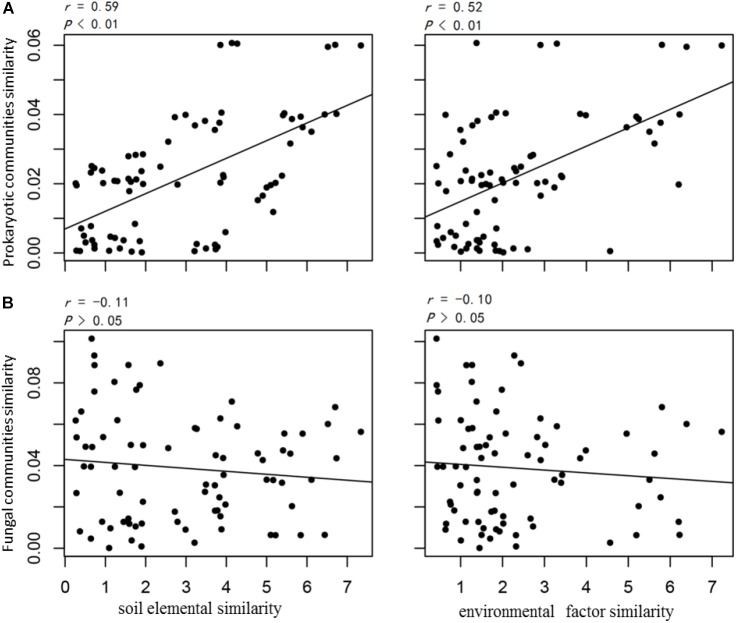
Pearson correlations between **(A)** the prokaryotic community and **(B)** the fungal community with soil elemental compositions and environmental attributes. Similarity values are directly indicated by calculated pairwise Euclid distances between samples.

**FIGURE 5:**
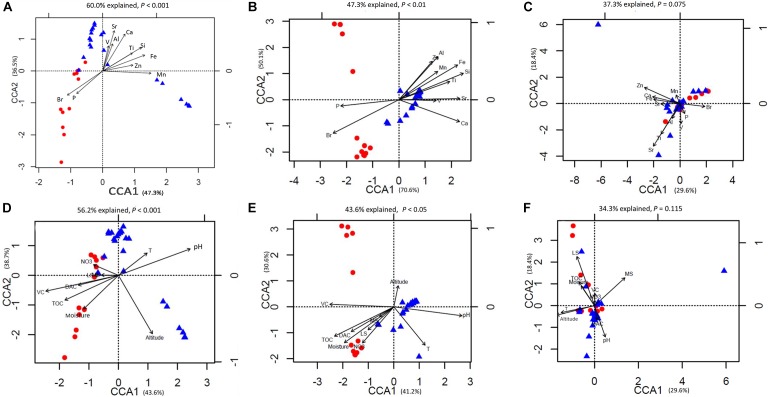
Canonical correspondence analysis (CCA) of **(A)** bacterial operational taxonomic unit (OTU) data and elemental compositions; **(B)** archaeal OTU data and elemental compositions; **(C)** fungal OTU data and elemental compositions; **(D)** bacterial OTU data and environmental attributes; **(E)** archaeal OTU data and environmental attributes; **(F)** fungal OTU data and environmental attributes.

The prokaryotic OTU composition showed a strong and significant correlation with soil elements (*r* = 0.59, *P* < 0.01, Pearson test) and a less but still significant correlation with environmental attributes (*r* = 0.52, *P* < 0.01, Pearson test; Figure 4A). The fungal community composition did not show significant correlations with either the soil elements or environmental attributes (*P* > 0.05; Figure 4B). The soil elements and environmental attributes in the CCA were selected by a variation inflation test (see the section “Materials and Methods” for details). The CCA results for bacterial and archaeal community compositions and soil elements, with significant models (both *P* < 0.01), indicated that the 11 soil elements were important factors controlling the bacterial and archaeal community structures, and these explained 60.0 and 47.3% of their variations, respectively (Figures 5A,B).

Among these elements, P and Br were important elements controlling the microbial community structures in Group 1, and the other lithophile and metal elements controlled the structures in Group 2. The importance of these soil elements was verified by a Monte Carlo test (*P* < 0.05, 999 permutations) with prokaryotic community data comprising bacterial and archaeal community compositions (Table 2). For fungal communities, the CCA analysis showed that the two groups were not well separated from the others, and the model was not significant (*P* > 0.05). Only 37.3% of the fungal community variations could be explained by the 11 soil elements (Figure 5C). Considering the relationships between microbial communities and environmental attributes, the same analysis was conducted with the CCA technique (Figures 5D–F). The models were significant between both bacterial (*P* < 0.01, 56.2% explained) and archaeal (*P* < 0.05, 43.6% explained) community structures and environmental attributes. The Monte Carlo test (999) revealed that TOC, soil pH, moisture, site altitude, DAC, and VC showed strong effects on prokaryotic communities. For fungal communities, the model was not significant within the confidence level (*P* > 0.05, 34.3% explained); however, soil pH, site altitude, MS, and LS were identified as environmental attributes that affected the fungal community composition (Monte Carlo test, *P* < 0.05, 999 permutations; Table 2). For the mantel test results for the microbial community structures, which included all factors investigated in this study, see Supplementary Table S3.

**Table 2:** Monte Carlo test of the factors (soil elemental compositions and environmental attributes) and compositions of microbial communities and phospholipid fatty acids (PLFA).

	Prokaryote		Fungi		PLFA	
	*r*^2^	*P*-value	*r*^2^	*P*-value	*r*^2^	*P*-value
**Soil elements**						
Al	0.2547	0.002**	0.0014	0.979	0.4648	0.001***
Ca	0.5647	0.001***	0.0012	0.981	0.3276	0.003**
Mn	0.5235	0.001***	0.0018	0.965	0.3859	0.001***
P	0.3372	0.005**	0.0488	0.378	0.2883	0.012*
Si	0.5621	0.001***	0.0299	0.609	0.6624	0.001***
Sr	0.5332	0.001***	0.2784	0.007*	0.4546	0.001***
Ti	0.3316	0.003**	0.2702	0.010*	0.3503	0.001***
V	0.2005	0.020*	0.0408	0.697	0.1828	0.041*
Zn	0.2309	0.009**	0.1120	0.216	0.3289	0.003**
Br	0.5059	0.001***	0.0369	0.558	0.6223	0.001***
Fe	0.5210	0.001***	0.0165	0.761	0.6978	0.001***
**Environmental factors**						
TOC	0.4139	0.001***	0.1748	0.099	0.6776	0.001***
NO_3_	0.0667	0.286	0.0055	0.792	0.5661	0.120
T	0.1531	0.068	0.1867	0.055	0.1042	0.176
pH	0.6958	0.001***	0.2385	0.007**	0.4830	0.001***
Moisture	0.2644	0.012*	0.1231	0.128	0.5844	0.001***
Altitude	0.3003	0.002**	0.2463	0.024*	0.0065	0.912
DAC	0.2007	0.030*	0.0454	0.516	0.3487	0.003**
MS	0.0320	0.557	0.2723	0.007**	0.0505	0.430
LS	0.0624	0.325	0.5927	0.001***	0.1367	0.103
VC	0.6465	0.001***	0.0298	0.779	0.4422	0.001***


*Significant differences (P < 0.05) are indicated in asterisk. ^∗∗∗^P < 0.001, ^∗∗^P < 0.01, ^∗^P < 0.05. P-values based on 999 permutations.*

### Microbial Biomass and Microbial Diversity Determined by the Phospholipid Fatty Acids (PLFA) Method

The total amounts of PLFA (totPLFA) of Group 1 were significantly higher than those of Group 2 (Wilcoxon test, *P* < 0.05; Supplementary Figure S3). The CCA analysis of the individual relative concentration (mol%) of the 45 most common PLFAs showed that, on the whole, the 11 soil elements and the 11 environmental attributes were all important factors controlling soil PLFA patterns (Supplementary Figure S4, *P* < 0.01), with 47.5 and 47.0% of the variations explained, respectively. Among these factors, each of the 11 elements and the pH, moisture, TOC, DAC, and VC representative of the environmental attributes had significant effects on the soil PLFA composition (Table 2). Microorganism categories including bacteria, fungi, and protozoa were classified by indictor PLFAs according to a microbial identification system (MIDI). The relative abundances of AM fungi, actinomycetes, and anaerobes were higher in Group 1, and the abundance of Gram-negative bacteria was higher in Group 2 (Supplementary Figure S5; Welch’ s *t*-test, two-sided, *P* < 0.05).

### Differences in the Microbial Community Composition Between the Two Groups

In our analysis, the classified mode of the random forests machine learning technique (Breiman, 2001; Cutler et al., 2007) could be accepted if the ratio of the baseline error to the observed error was greater than 2, and we considered an OTU to be highly predictive if its importance score was at least 0.001. For bacteria, random forest analysis revealed that 58 OTUs distinguished the two groups; Acidobacteria were overrepresented in Group 1, and the OTUs assigned to the Thermoleophilia class of the phylum Actinobacteria, and the genus *Geobacillus* of the phylum Firmicutes were overrepresented in Group 2. For archaea, 38 OTUs distinguished the two groups, which included all of the OTUs except for the 12 OTUs with no assigned taxa. Some 11 OTUs were overrepresented in Group 1, and 17 OTUs were overrepresented in Group 2; all were assigned to the genus *Candidatus Nitrososphaera* of the phylum *Crenarchaeota*. As the ratio of the baseline error to the observed error of the random forest analysis with fungal OTUs was less than 2, we considered that the non-obvious classified results suggested that there was no credible difference in the fungal community composition between the two groups (Supplementary Table S4).

## Discussion

### Microbial Community Distribution in the Fildes Region

Compared to culturable and traditional molecular techniques, the Miseq sequencing method is able to identify a great number of special molecular labels in organisms, and thus, it provides in-depth data to analyze soil microbial diversity. In our study, five bacterial phyla (Actinobacteria, Acidobacteria, Proteobacteria, Chloroflexi, and Gemmatimonadetes), two archaeal phyla (Crenarchaeota and Euryarchaeota), and three fungal phyla (Ascomycota, Basidiomycota, and Zygomycota) were commonly detected in the soil samples. These results were somewhat different from those of previous studies on terrestrial bacterial compositions in Antarctica. For example, Bacteriodetes was abundant at sites in the Ellsworth Mountains, Victoria Land, and Signy Island (Aislabie et al., 2006; Yergeau et al., 2007b; Niederberger et al., 2008; Chong et al., 2010), but it was much less abundant (0.2–13.1%) in all of the samples in this study. Moreover, Verrucomicrobia was a “rare” member (0–1.5%) in our quadrat plots, but it was found to be more abundant in Miers Valley (5%); meanwhile, it was found to be completely absent from mineral soils in the Antarctic Dry Valleys region (Smith et al., 2006; Lee et al., 2012). As the quadrats in our study all had hairgrass growth, vegetation may be one of the main causes for these differences. Thus, our results were similar to the bacterial community compositions in other vegetated parts of Antarctica, with relatively high abundances of Chloroflexi and Gemmatimonadetes, which have strong reported relationships with plants (Castro et al., 2010; Banning et al., 2011; DeBruyn et al., 2011). Previous studies of Antarctic archaeal communities were mostly concentrated in marine and lake environments (Delong et al., 1994; Murray et al., 1998; Church et al., 2003; Karr et al., 2006; Kalanetra et al., 2009), and in this study, only two archaeal phyla (Crenarchaeota and Euryarchaeota) were detected, with Crenarchaeota representing the overwhelming majority (>90%) of the archaeal communities. This was consistent with other terrestrial archaeal structures of Antarctica derived by using other investigative methods [e.g., clone libraries of rRNA genes and microarrays (Yergeau et al., 2009; Ayton et al., 2010)]. To the best of our knowledge, no study has investigated the fungal community structure in terrestrial ecosystems of maritime Antarctica by using high-throughput sequencing. We found that Ascomycota, Basidiomycota, and Zygomycota were the predominant fungal phyla in all samples, and these have been revealed as the most common phyla presenting in other Antarctic areas with other methods [e.g., cultured isolation, denaturing gradient gel electrophoresis (DGGE), and PCR amplification techniques] (Arenz et al., 2006; Malosso et al., 2006; Bridge and Newsham, 2009; Rao et al., 2012). In this study, *Aspergillus* (0.04–48.0%) and *Pseudogymnoascus* (0.07–40.5%) were the most common fungal genera in the samples. Although most species of *Aspergillus* are thermophilic, their spores are known to be able to survive under extreme conditions for decades (Robinson, 2001). *Pseudogymnoascus* has a wide distribution and occurs in the soils of Arctic, alpine, temperate, and Antarctic regions; species in this genus have the ability to colonize and utilize different carbon sources and can increase in abundance at lower temperatures (Gomes et al., 2018). Furthermore, a small number of OTUs were classified into Rozellomycota, Glomeromycota, and Chytridiomycota (<3%, each phylum), and in total, 222 fungal families had been found. The diversity of fungi in the Antarctic soil was beyond our imagination.

### The Prokaryotic Community Composition of the Quadrats Can Be Divided Into Two Groups

The NMDS analysis showed that the bacterial and archaeal community compositions of the quadrats can be divided into two groups, but this was not possible for the fungal community (Figure 3). Random forest analysis revealed that the OTUs belonging to Alphaproteobacteria, Acidobacteria, and Bacteroidetes were mostly overrepresented in Group 1. These phyla have shown positive correlations with vegetation and the rhizosphere in farmland, Arctic glacier moraines, and the Brazilian Antarctic Station (Gomes et al., 2018). Alphaproteobacteria are among the most abundant marine cellular organisms (Williams et al., 2007). The results of our study showed different patterns at the family level, with Acidobacteriaceae, Koribacteraceae, Chitinophagaceae, and Rhodospirillaceae as the most overrepresented families in Group 1. The family level differences from our study could have been due to the locations of sampling points and the diverse sequencing methods used. Acidobacteriaceae and Koribacteraceae belong to the phylum Acidobacteria, and very little is known about these two families or what their roles are in the environment because of the lack of isolates representing the families. However, most members of this family can be found in and isolated from acidic environments (Jones et al., 2009). The family *Rhodospirillaceae* produce energy through photosynthesis and are often found in anaerobic aquatic environments. These overrepresented taxa may have been related to the habitats of Group 1, which had higher vegetation coverage, lower pH values, and beach and sedimentary landforms. Conversely, in Group 2, the major overrepresented OTUs were in the class Thermoleophilia of the phylum Actinobacteria. Thermoleophilia is a newly proposed class of the phylum Actinobacteria that was created following the splitting of Rubrobacteridae (Ludwig et al., 2010), and its ecological position is not well understood. However, Thermoleophilia is abundant in deserts and glacier forelands (Crits-Christoph et al., 2013; Zhang et al., 2016); moreover, some isolated cells were found to be culturable in low nutritional media during long incubation periods. Thus, it is reasonable that this class was found in the quadrats located in the volcanic stratigraphy with high proportions of lithospheric elements and low nutrient conditions. In addition, we also found that five OTU sequences affiliated with Flavobacteriaceae extracted from Group 1 were clustered in a marine clade, and no marine clade OTUs of Flavobacteriaceae were found in Group 2 (Supplementary Figure S6). Members of the family Flavobacteriaceae (marine clades) are among the most abundant picoplankton in coastal and polar oceans, and a number of genera have potential evolutionary sources from the ocean (Bowman, 2006). Regarding the genus *Candidatus Nitrososphaera*, the vital ammonia-oxidizing archaea (Spang et al., 2012; Swanson and Sliwinski, 2013) was an overrepresented archaeal OTU in both groups. The uncultivable species SCA1170 of the genus *Candidatus Nitrososphaera* was a major genus in Group 2 but it did not appear in Group 1. This, along with evidence from the NMDS analysis, implies that the two different landforms have diverse archaeal communities. For fungal communities, no credible difference was found between the two groups according to the ratio of baseline error to observed error (<2), and this was in agreement with the PERMANOVA tests that showed that there was no significant difference in the fungal community (*Pseudo-F* = 1.3, *P* = 0.196). These results could be explained by the reasoning that fungal vectoring can occur over long distances due to atmospheric circulation and transport by birds and animals, and many species of fungi have great dispersal potential as confirmed, for the Antarctic Peninsula related to specific weather events, by spore trap data (Marshall, 1996). However, this does not mean that the distribution of soil fungi in our study was entirely random. An earlier study found that the fungal community structure can be influenced by the type of vegetation cover along latitude in Antarctica (Yergeau et al., 2007a); our study results indicated that the elements Sr and Ti, soil pH, site altitude, and number of moss species and lichen species could significantly affect the fungal community composition mostly over small-scale regions. Lichen and moss species numbers were the strongest environmental correlation factors for the fungal communities. It was previously reported that some fungal species coexist with moss and lichen in Antarctica (Tosi et al., 2002; Lee et al., 2008).

### Relationship Between the Microbial Community Distribution and Historical Contingencies in the Fildes Region

Historical contingency causes the effect of the order and timing of past events on community assembly (Fukami, 2015). Events that can cause historical contingency effects can be either abiotic or biotic. Examples of abiotic events are disturbances such as floods, fires, storms, and earthquakes that initiate changes in the community assembly (Crawley, 2004). In the study area, a series of geological events including volcanic activity, glacial erosion and retraction, isostatic uplift, and sea level change have created rich landform types. According to geomorphological and sedimentary evidence, the relative sea level (RSL) gradually fell to <14.5 m between 7000 and 4750 cal a BP as a consequence of isostatic uplift in response to regional deglaciation (Ingolfsson et al., 1998; Watcham et al., 2011). During landform formation, rich marine elements and nutrients were transferred to the younger land (Group 1, approximately 4300 years BP) (Boelter, 2011; Michel et al., 2014). Moreover, from approximately 2500 years ago, mammals, especially penguins, began to colonize the newly uplifted beaches until at least ∼500 years ago when the raised beaches were abandoned [according to chronological research of abandoned rookeries on King George Island (Tatur et al., 1997)]. These abandoned penguin rookeries are indicators of the Holocene paleoclimate, and these are also sites where rich nutrients accumulated during the active period (Baroni and Orombelli, 1994). This input of elements, nutrients, and marine microorganisms clearly promoted the development of soil and plant growth and influenced the patterns of microbial community formation.

**FIGURE 6:**
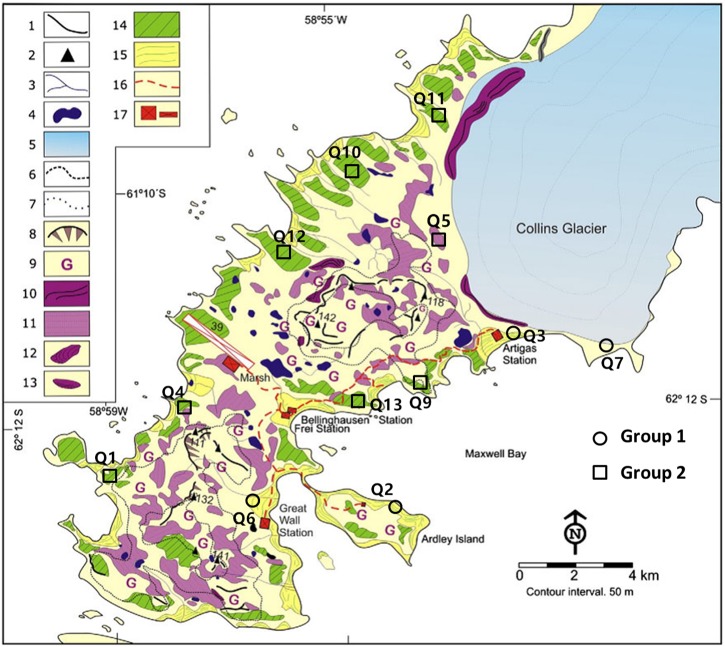
Geomorphological map of the Fildes Region derived from Michel et al. Quadrats of Group 1 and Group 2 were located at Holocene raised beaches (No. 15) and marine platforms (periglacial landforms belonging to Tertiary volcanic stratigraphy, No. 14). The landform type of quadrat Q7 can be deemed as part of the Holocene raised beaches because it suffered recent glacio-isostatic uplift but was still covered by ice during that uplift (personal communication with Michel, 2018). Please refer to original literature for landforms marked by other numbers. Reprinted from Michel et al. (2014), with permission from Elsevier.

In this study, the prokaryotic community composition of the quadrats was divided into two groups that were correlated with the soil elemental compositions and environmental attributes. Interestingly, a published geologic map of the Fildes Region and previously reported literature (Liu and Zheng, 1988; Michel et al., 2014) showed that the quadrats in Group 2 were located in Tertiary volcanic stratigraphy and those in Group 1 were located on Holocene raised beach (Figure 6). We suggest that there is a relationship between microbial distributions and historical contingencies (the development of landforms) at this small spatial scale. The prokaryotic communities were characterized by high biomasses, diversity, and marine related compositions, but were more volatile in the younger geological layers within a transboundary ecological stage from ocean to land. The evolution of the microbial communities related to historical contingencies will be interesting to research further with long-term monitoring data. Under the same extreme conditions in Antarctic, the microbial distributions in the region may become similar. However, different evolutionary paths can lead to similar fitness states in a prevailing environment but very different fitness states in other environments (Jablonski, 1986). So, the subtle differences in microbial communities resulting from historical contingencies between the groups may lead to different responses and evolutionary outcomes during future climate and environmental change. If the responses are the same in the two historical contingency related groups, this will mean that the microbial community evolution is relatively path independent; conversely, different responses will mean that the evolution is path dependent. Evaluating the deterministic factors during evolution may contribute to better predictions of responses during future changes.

### Factors That Determined the Microbial Community Distribution

Soil elemental profiles can be seen as proxy indicators of soil types and landforms with strong soil–landform relationships (Lark, 1999; Evans, 2012). In previous studies, soil compositions tested by X-ray fluorescence spectrometry have been revealed to be the key factors for the distribution of bacterial and fungal communities at some field sites within sediments, glacier forefields, and deserts (Handley et al., 2010; Summers et al., 2013; Meola et al., 2014; Goncalves et al., 2016). In this study, the CCA analysis and similarity test results showed that both environmental attributes and soil elemental compositions could influence the microbial structure and biomass (totPLFA, nmol/g soil dry weight). However, compared with environmental attributes, the relationship between the soil elemental composition and prokaryotic community was stronger (Figures 4, 5). Mantel analysis revealed that the relative abundance of almost every element was important for shaping prokaryotic compositions (Supplementary Table S3). The quadrat plots located on the Holocene raised beach landform showed relatively high abundances of P, S, Cl, and Br, which were more correlated to marine environments and organisms. These elements are readily absorbed by vegetation and microorganisms, and presumably, these elements influenced the development of microbial community structures in Group 1 (CCA analysis; Figure 4). The accumulation of elements P and S may represent not only marine inputs, but also mammal and bird excrement that accumulated in these raised beaches during the early stage of uplifted landform formation (Tatur et al., 1997). Meanwhile, the halogen elements Cl and Br in island coastal soil were likely derived mostly from seawater (Santos et al., 2007).

Conversely, the bacterial and archaeal community compositions of Group 2 were more correlated with lithophile-elements (Si, Al, Ca, Sr, Ti, V, and Fe, CCA analysis), and the landforms were almost completely isolated from the external environment until the icecap retreated ∼11,000–7500 cal a BP (Watcham et al., 2011). This suggests that soil in the quadrats located in tertiary volcanic stratigraphy mainly developed from the chemical and biological weathering of volcanic rock generated by Tertiary volcanism, and it underwent paraglacial and periglacial processes. That may explain the lower soil biomass, nutrient concentrations, and vegetation coverage as compared with Group 1; the limited nutrient input distinguished the prokaryote community composition from that of the nutrient-rich soil of Group 1. Therefore, we believe that the elemental composition of the soil associated with these landforms reveals important geological background information and historic effects.

In Group 1, the soil contained high contents of TOC, NH_4_^+^, and NO_3_^-^, and the vegetation coverage was high; these factors were correlated with the prokaryotic community. The relatively low pH values (Supplementary Table S1) may have been the result of the higher vegetation coverage and more humus and fulvic acids produced by mosses and lichens (Fabiszewski and Wojtun, 1993). While the rich nutrients and elements transferred from Holocene raised beach marine environments could have promoted soil development and plant growth, these environmental attributes seemed to be secondary factors affecting the prokaryotic community when compared to soil elemental compositions. Unlike Group 1, very small amounts of nutrients in the soil samples of Group 2 were more likely caused by current precipitation, snowfall, and animal activity. In keeping with reported studies (Fierer and Jackson, 2006; Lauber et al., 2009; Dumbrell et al., 2010), pH was one of the most influential factors affecting the distribution of microbial communities in this study. Following the comparisons of the microbial diversity data from high-throughput sequencing, the explained variations of soil PLFA patterns in the CCA for both soil elements (47.5%) and environmental attributes (47.0%) were found to be less than the explained variations of bacterial OTUs (60.0 and 56.2%, respectively) (Figure 4 and Supplementary Figure S4). Since PLFAs were extracted not only from bacterium, but also from fungi and even protozoa (Zelles, 1999), the potential movability of fungi and protozoa may explain the differences between the two analysis methods.

Interestingly, both prokaryote and fungal communities were significantly correlated to the altitude of the sample location. Despite the slightly different altitudes (ranging from 11 to 56 m), there were no significant changes in temperature, oxygen content, etc., which seems to suggest that geological uplift had an impact on the microbial communities. We also noted that the soil elemental compositions and environmental attributes of ancient landforms investigated in our study were relatively stable, while those of younger landforms were more volatile (from Euclidean distances computed between samples from the PCA analysis in Figure 1A and Supplementary Table S5). This suggests that the quadrat plots of Group 1 may be in an unstable new geological layer within a transboundary ecological stage from ocean to land, and disturbance from the new terrestrial environment may increase the heterogeneity of the geomorphic ecology.

### Why the Historical Effects of Geological Evolution on the Microbial Distribution Can Be Highlighted in the Fildes Region

The importance of geological factors such as landforms and lithology on microbial structures is not well understood (Viles, 2012). Locations with distinct geologic factors generally exhibit geographical isolation; hence, they are mostly distributed over large and global scales. Limited research has shown that different landforms and soil profiles can be important drivers of bacterial diversity at the regional scale (>1000 km distance), and their impacts can be more significant than contemporary environmental factors (Ge et al., 2008; Reith et al., 2012). Interestingly, we found that on such a small spatial scale, prokaryotic communities also showed a landform-governed distribution trend, and the microbial community structure was expected to be an indicator of the formation of the landform. The role of geological evolution in the microbial distribution can be highlighted in this study area because of the following features. (i) There is clear evidence of geological evolution in the Fildes Region in maritime Antarctica, as documented in the relatively sufficient geological literature; glacial activity, sea level changes, and tectonic uplift due to climate change after the LGM have all resulted in landform heterogeneity at a small spatial scale. (ii) Seasonal freezing–thawing cycles in the area have enhanced soil development and promoted soil particle and nutrient migration to the upper and surface soil layers. (iii) The low activity of microorganisms under the cold climate conditions and the minimal human disturbance where the quadrats were established have resulted in the maintenance of relatively stable microbial community diversity for long periods of time after the geological changes. For these reasons, these ice-free regions in maritime Antarctica represent suitable research sites for studying the influence of geomorphological features on microbial community distributions; other such suitable sites may be present in the ice-free regions of Antarctica as well.

## Conclusion

In this study, we observed differences between prokaryotic communities linked to the effect of the investigated abiotic factors, and these differences reflect the sites division according to landform type. This study provides evidence for the influence of geological evolutionary processes on the small-scale distribution of microbial communities. Specifically, the microbial community structure was found to be related to two different types of land in the Fildes Region, King George Island. In addition, other locations in Antarctica have experienced the same type of glacial activity and isostatic uplift as the coastal ice-free areas around King George Island, maritime Antarctica, and Prince Charles Mountains area and East Antarctica (Wellman and Tingey, 1981), which implies that microbial communities in these areas may be diverse and show the influence of different geological evolutionary events at small to moderate spatial scales. Continued research, which is already in progress, will verify whether microbial communities can be used as indicators of different landforms in other similar geological areas in maritime Antarctica. This research will also contribute to our understanding of different microbial communities in limited spatial regions based on geological research, and support possibility of a site-specific landform assignment through the analysis of the soil prokaryotic community structure.

## Author Contributions

YZ, XG, and FP designed the experiment and supervised all work. LL was involved in the experiment of PLFA analysis. XC, YZ, YY, CL, SC, and QZ were involved in the field work. FJ and YZ supported the sequencing data analysis. All authors contributed to the preparation and revision of the manuscript.

## Conflict of Interest Statement

The authors declare that the research was conducted in the absence of any commercial or financial relationships that could be construed as a potential conflict of interest.
