# Supplementary figures and images for: Small-Scale Soil Microbial Community Heterogeneity Linked to Landform Historical Events on King George Island, Maritime Antarctica
Source: Front Microbiol. 2018 Dec 10;9:3065. doi: 10.3389/fmicb.2018.03065 (PMC6296293; doi:10.3389/fmicb.2018.03065)

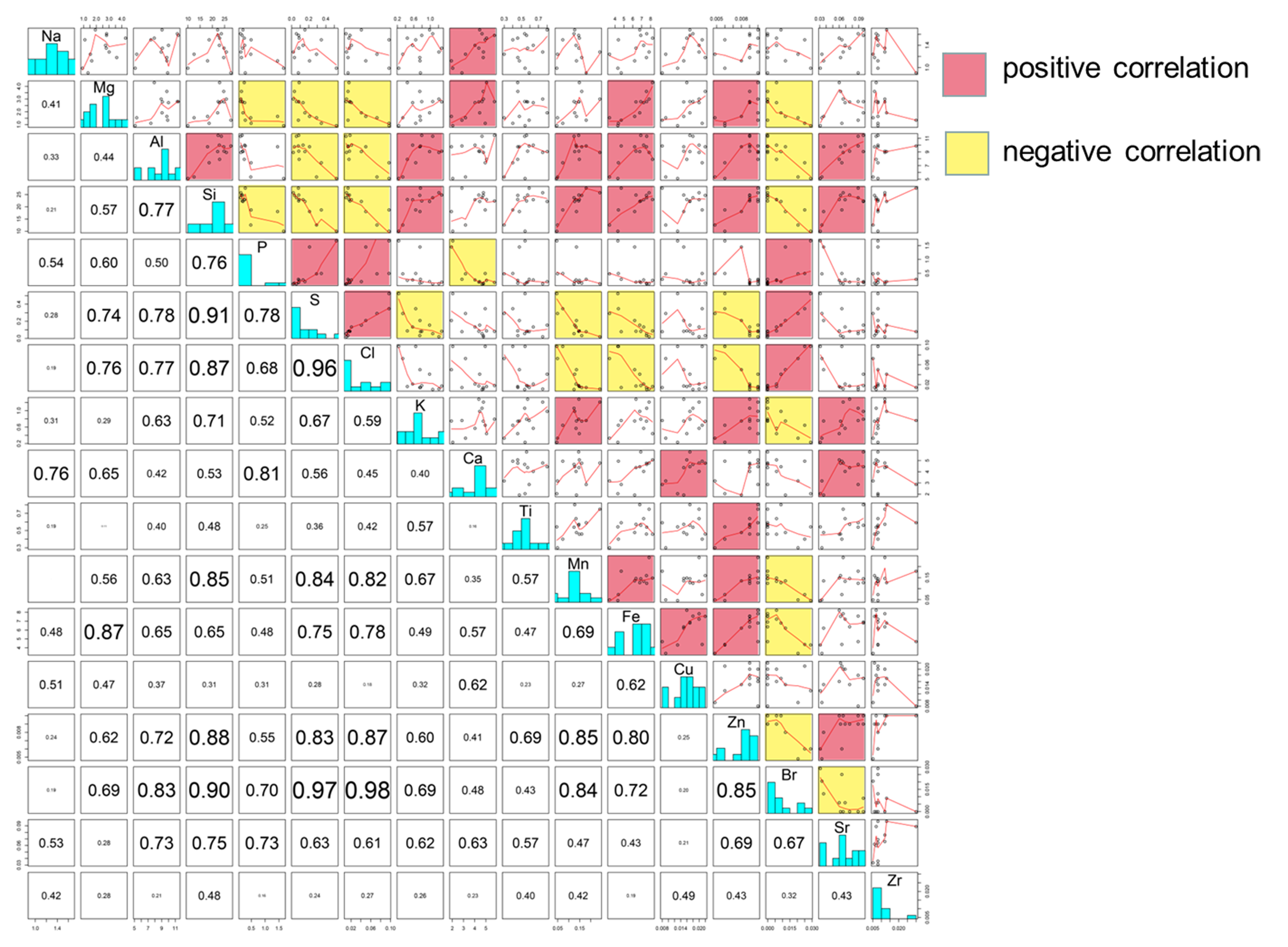

Supplement: Supplementary file 1 [file Image_1.TIF]

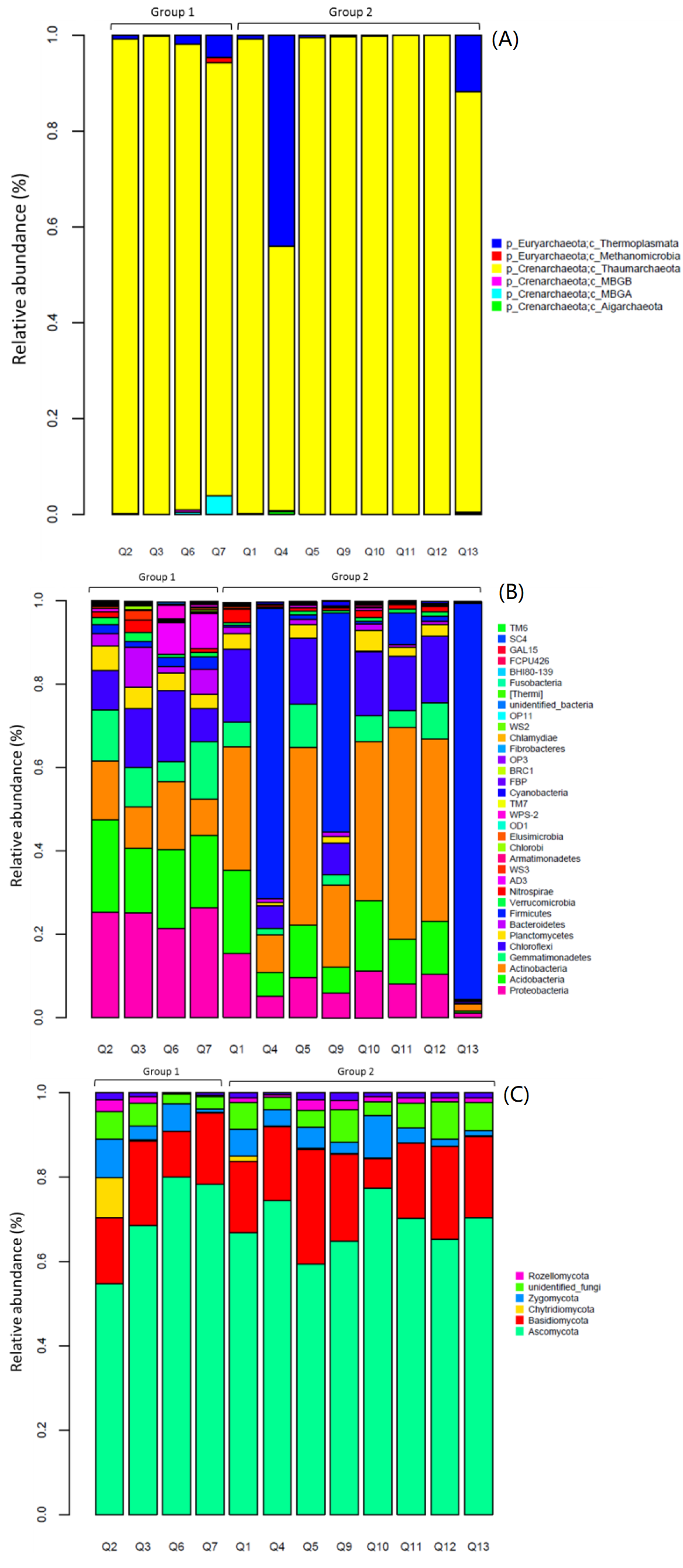

Supplement: Supplementary file 2 [file Image_2.TIF]

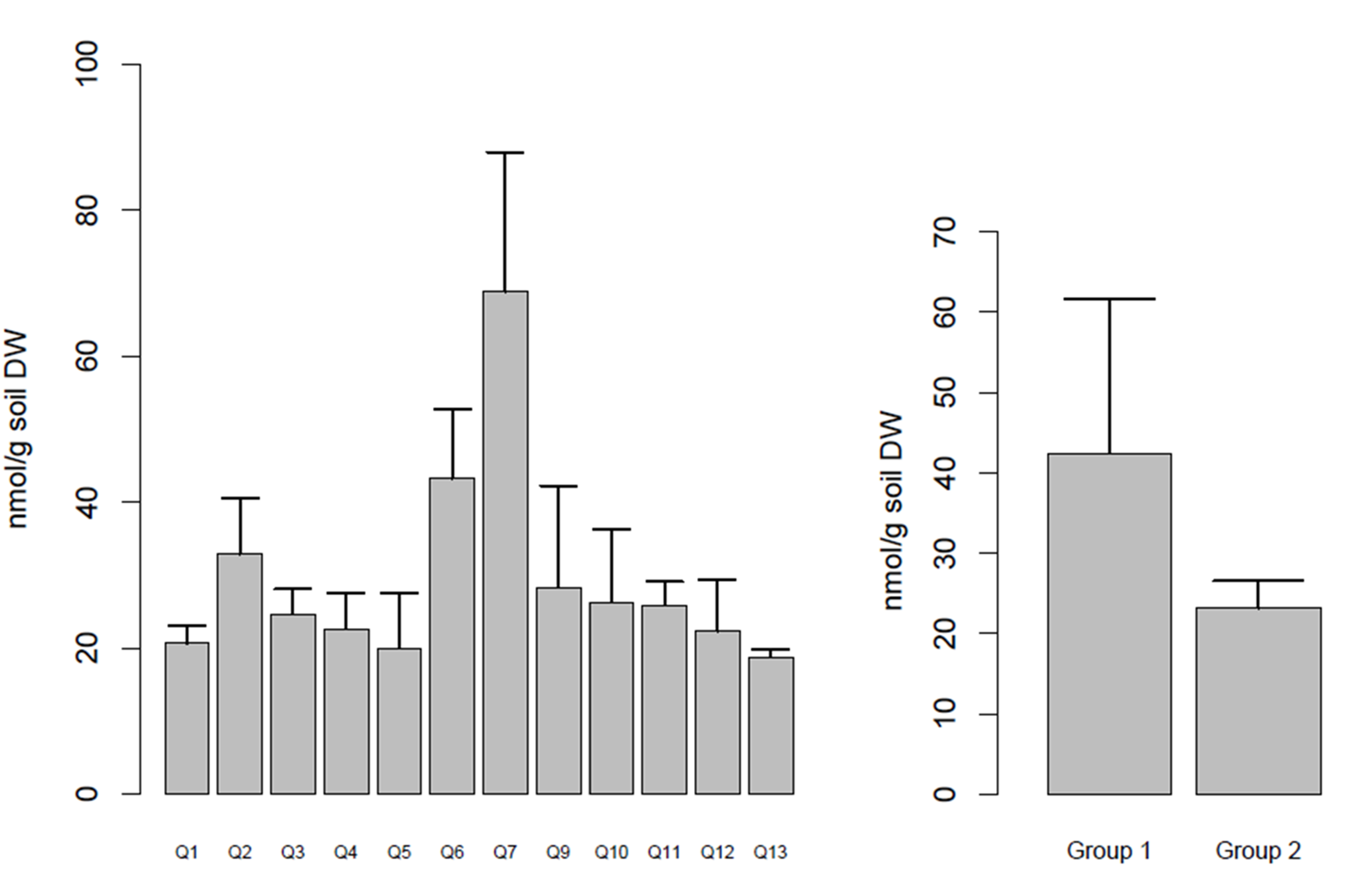

Supplement: Supplementary file 3 [file Image_3.TIF]

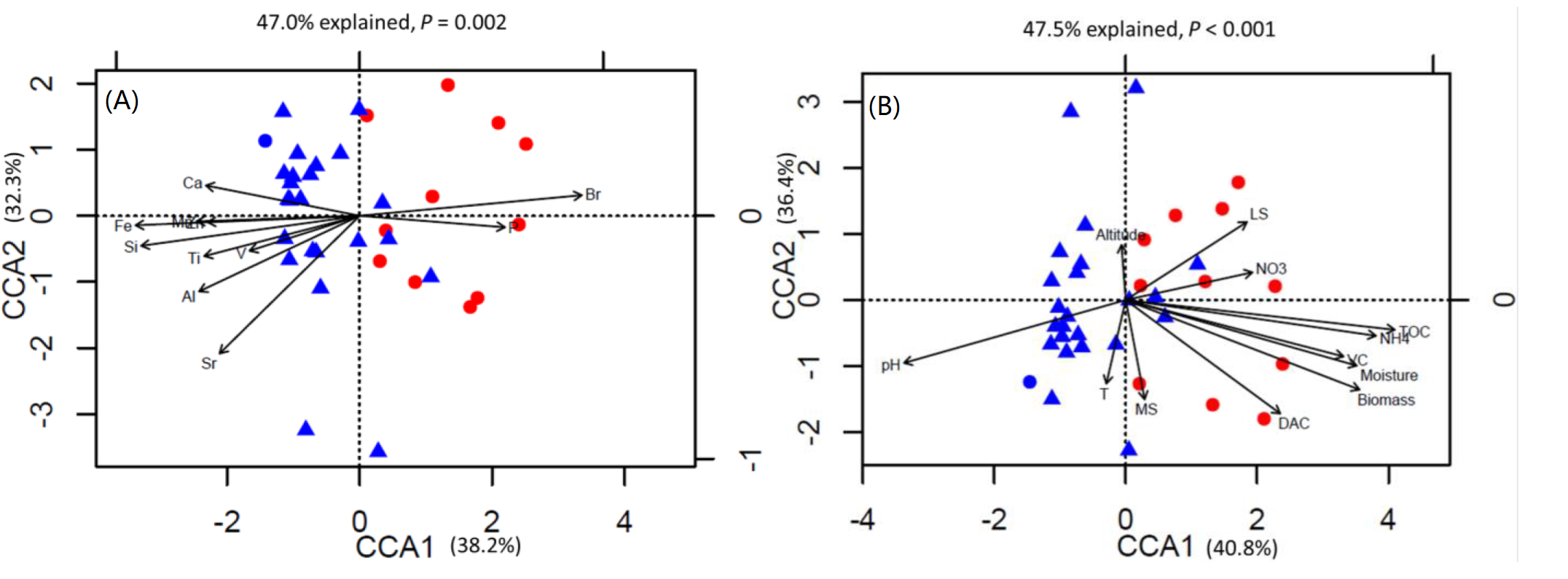

Supplement: Supplementary file 4 [file Image_4.TIF]

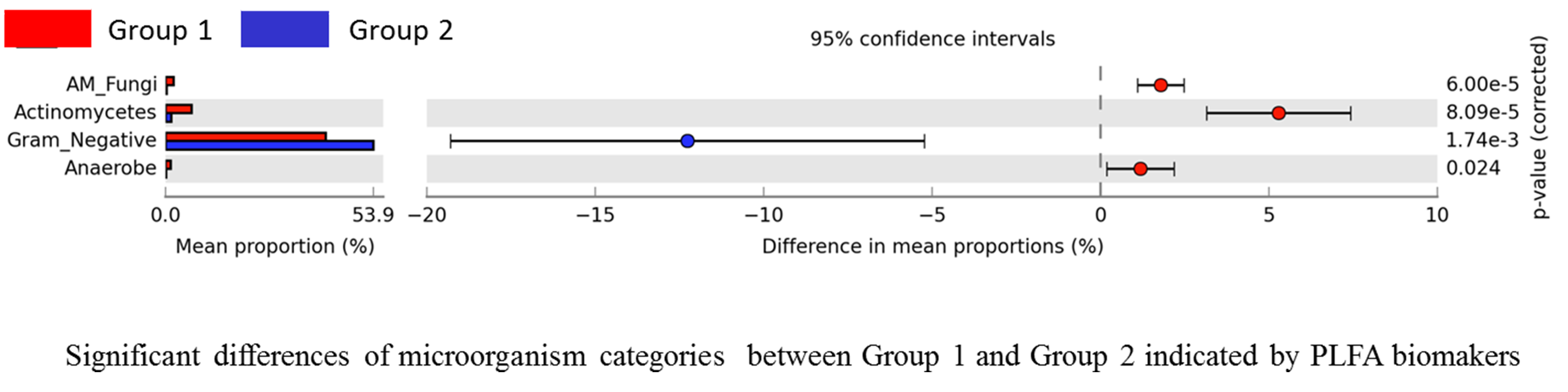

Supplement: Supplementary file 5 [file Image_5.TIF]

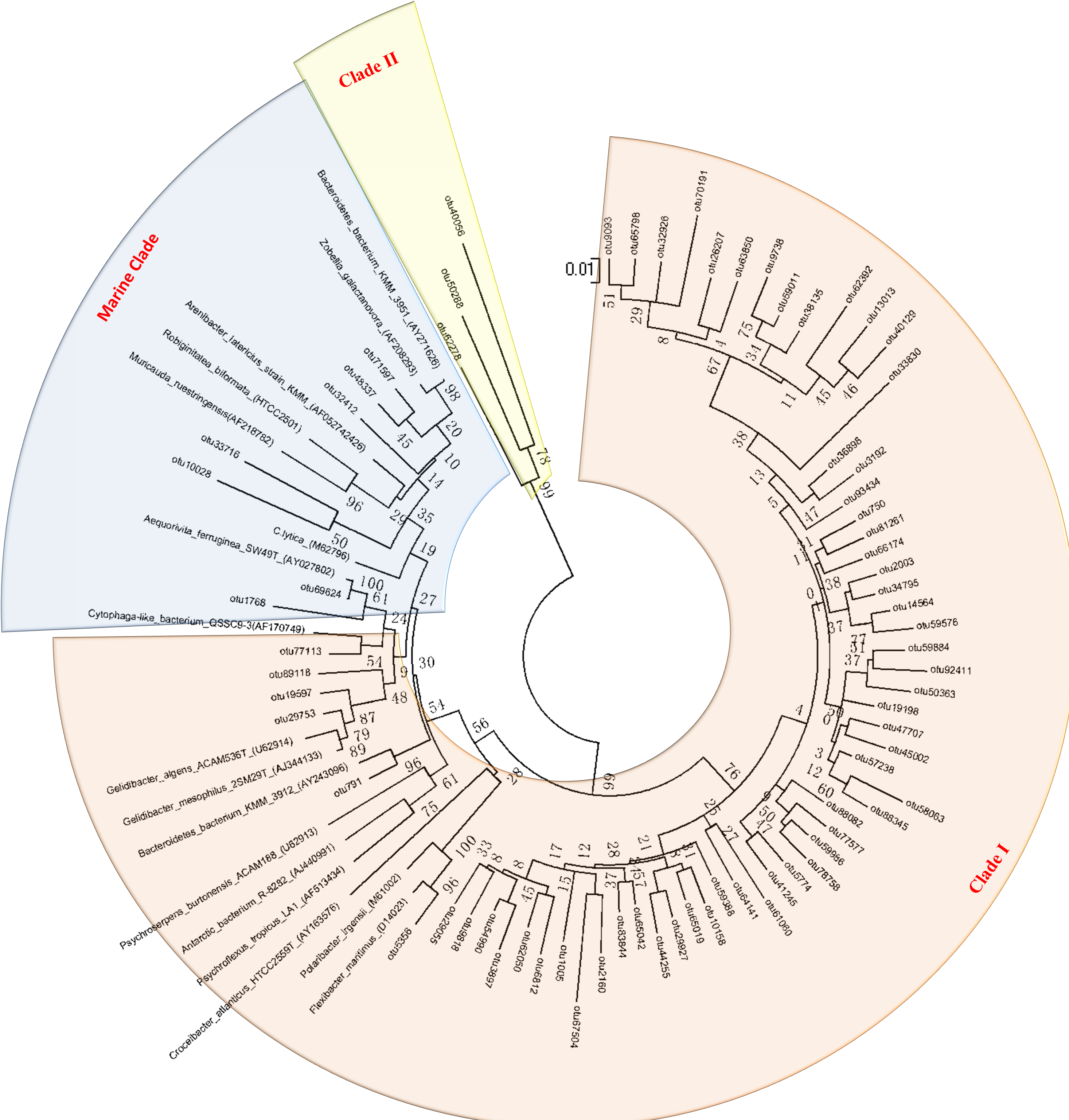

Supplement: Supplementary file 6 [file Image_6.TIF]

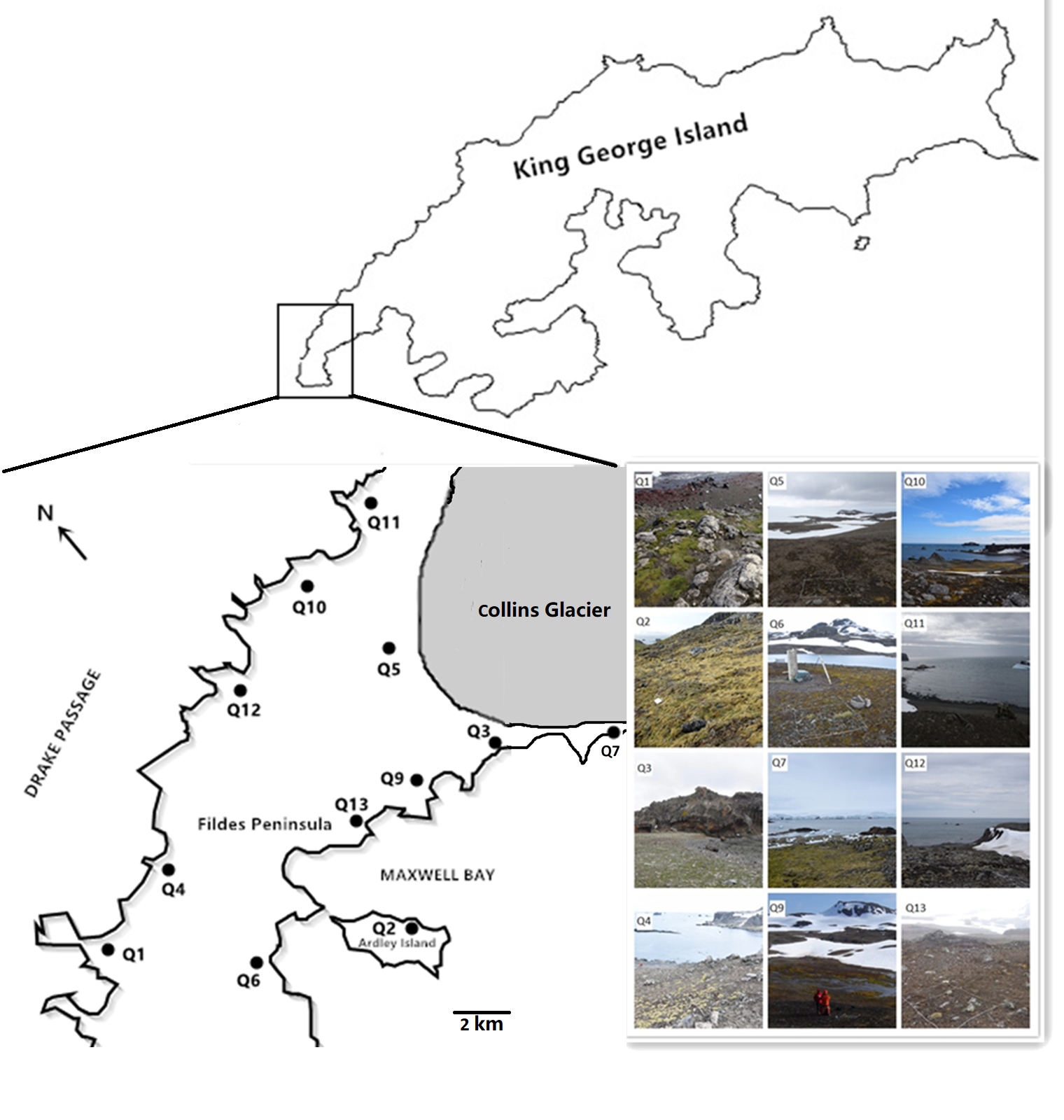

Supplement: Supplementary file 7 [file Image_7.TIF]
