# Supplementary material for: Small-Scale Soil Microbial Community Heterogeneity Linked to Landform Historical Events on King George Island, Maritime Antarctica
Source: Front Microbiol. 2018 Dec 10;9:3065. doi: 10.3389/fmicb.2018.03065 (PMC6296293; doi:10.3389/fmicb.2018.03065)
Supplement: Supplementary file 8 [file Table_1.DOCX]

| **Factor** | **Quadrat plots** | | | | | | | | | | | |
| --- | --- | --- | --- | --- | --- | --- | --- | --- | --- | --- | --- | --- |
|  | **Q1** | **Q2** | **Q3** | **Q4** | **Q5** | **Q6** | **Q7** | **Q9** | **Q10** | **Q11** | **Q12** | **Q13** |
| Al (%)** | 8.19 (0.12) | 6.21 (2.55) | 8.55 (0.94) | 9.69 (0.25) | 9.65 (0.55) | 9.54 (0.27) | 6.54 (1.33) | 9.37 (0.13) | 10.22 (0.13) | 11.23 (0.20) | 11.27 (0.11) | 8.32 (0.55) |
| Ca* | 5.01 (0.17) | 2.17 (0.47) | 5.50 (0.54) | 5.03 (0.17) | 4.28 (0.25) | 1.65 (0.21) | 3.27 (0.84) | 4.62 (0.21) | 4.24 (0.14) | 4.40 (0.29) | 5.22 (0.02) | 4.09 (0.59) |
| Cl*** | 0.02 (0.00) | 0.07 (0.01) | 0.05 (0.02) | 0.00 (0.00) | 0.01 (0.01) | 0.06 (0.01) | 0.04 (0.01) | 0.01 (0.01) | 0.02 (0.00) | 0.01 (0.01) | 0.02 (0.00) | 0.00 (0.00) |
| Cu | 0.01 (0.00) | 0.02 (0.00) | 0.02 (0.00) | 0.02 (0.00) | 0.02 (0.00) | 0.02 (0.00) | 0.01 (0.00) | 0.02 (0.00) | 0.02 (0.00) | 0.02 (0.00) | 0.02 (0.00) | 0.02 (0.00) |
| Fe*** | 7.87 (0.30) | 4.95 (1.44) | 4.61 (0.50) | 7.62 (0.20) | 7.28 (0.56) | 6.26 (0.30) | 5.13 (1.11) | 7.03 (0.35) | 7.50 (0.22) | 7.73 (0.26) | 7.26 (0.11) | 9.10 (1.18) |
| K*** | 0.43 (0.02) | 0.28 (0.10) | 0.68 (0.04) | 0.78 (0.04) | 1.23 (0.05) | 0.76 (0.04) | 0.38 (0.08) | 1.25 (0.10) | 0.61 (0.01) | 1.06 (0.06) | 0.82 (0.04) | 1.16 (0.10) |
| Mg*** | 4.31 (0.26) | 0.93 (0.20) | 1.33 (0.15) | 3.43 (0.26) | 2.13 (0.11) | 1.61 (0.10) | 1.79 (0.51) | 3.00 (0.26) | 2.82 (0.03) | 2.57 (0.02) | 2.74 (0.08) | 3.81 (0.63) |
| Mn*** | 0.14 (0.01) | 0.06 (0.03) | 0.09 (0.01) | 0.15 (0.04) | 0.14 (0.02) | 0.13 (0.03) | 0.07 (0.03) | 0.16 (0.01) | 0.14 (0.00) | 0.12 (0.00) | 0.14 (0.00) | 0.28 (0.09) |
| Na | 1.68 (0.05) | 0.85 (0.37) | 1.67 (0.18) | 1.32 (0.14) | 1.65 (0.15) | 1.09 (0.12) | 1.38 (0.31) | 1.49 (0.08) | 1.49 (0.04) | 1.57 (0.10) | 1.55 (0.05) | 0.98 (0.34) |
| P*** | 0.23 (0.02) | 3.07 (1.69) | 0.48 (0.09) | 0.22 (0.09) | 0.19 (0.02) | 1.74 (0.74) | 1.07 (0.21) | 0.14 (0.00) | 0.22 (0.03) | 0.11 (0.01) | 0.15 (0.01) | 0.12 (0.01) |
| S*** | 0.05 (0.00) | 0.48 (0.19) | 0.25 (0.08) | 0.03 (0.01) | 0.08 (0.04) | 0.22 (0.02) | 0.42 (0.20) | 0.02 (0.01) | 0.10 (0.02) | 0.04 (0.00) | 0.07 (0.01) | 0.01 (0.01) |
| Si*** | 23.88 (0.13) | 10.38 (2.68) | 20.28 (1.75) | 24.68 (0.56) | 24.31 (0.63) | 17.83 (0.71) | 17.06 (3.50) | 25.83 (0.19) | 23.56 (0.55) | 24.28 (0.11) | 23.25 (0.23) | 25.43 (0.25) |
| Sr*** | 0.06 (0.00) | 0.04 (0.01) | 0.07 (0.00) | 0.07 (0.00) | 0.11 (0.01) | 0.04 (0.00) | 0.04 (0.01) | 0.08 (0.01) | 0.07 (0.00) | 0.08 (0.00) | 0.09 (0.01) | 0.06 (0.01) |
| Ti*** | 0.51 (0.03) | 0.45 (0.12) | 0.41 (0.04) | 0.48 (0.01) | 0.72 (0.04) | 0.45 (0.03) | 0.57 (0.14) | 0.61 (0.01) | 0.63 (0.03) | 0.56 (0.03) | 0.65 (0.02) | 0.77 (0.03) |
| V* | 0.02 (0.02) | 0.01 (0.01) | 0.02 (0.01) | 0.01 (0.02) | 0.02 (0.02) | 0.01 (0.01) | 0.00 (0.00) | 0.01 (0.01) | 0.01 (0.02) | 0.03 (0.00) | 0.01 (0.02) | 0.02 (0.02) |
| Zn** | 0.01 (0.00) | 0.01 (0.00) | 0.01 (0.00) | 0.01 (0.00) | 0.01 (0.00) | 0.01 (0.00) | 0.01 (0.00) | 0.01 (0.00) | 0.01 (0.00) | 0.01 (0.00) | 0.01 (0.00) | 0.01 (0.00) |
| Zr | 0.01 (0.00) | 0.01 (0.00) | 0.01 (0.00) | 0.01 (0.00) | 0.01 (0.00) | 0.01 (0.00) | 0.01 (0.00) | 0.01 (0.00) | 0.01 (0.00) | 0.01 (0.00) | 0.01 (0.00) | 0.01 (0.00) |
| Ni | 0.00 (0.00) | 0.00 (0.00) | 0.00 (0.00) | 0.00 (0.00) | 0.00 (0.00) | 0.00 (0.00) | 0.00 (0.00) | 0.00 (0.00) | 0.00 (0.00) | 0.00 (0.00) | 0.00 (0.00) | 0.00 (0.00) |
| Br*** | 0.00 (0.00) | 0.03 (0.01) | 0.02 (0.00) | 0.00 (0.00) | 0.00 (0.00) | 0.01 (0.00) | 0.02 (0.00) | 0.00 (0.00) | 0.00 (0.00) | 0.00 (0.00) | 0.00 (0.00) | 0.00 (0.00) |
| Cr | 0.00 (0.00) | 0.01 (0.00) | 0.00 (0.01) | 0.00 (0.00) | 0.00 (0.00) | 0.00 (0.00) | 0.00 (0.00) | 0.00 (0.01) | 0.00 (0.00) | 0.00 (0.00) | 0.00 (0.00) | 0.00 (0.00) |
| TOC (mg/kg)*** | 0.74 (0.05) | 16.89 (8.00) | 7.00 (2.25) | 0.36 (0.16) | 0.77 (0.53) | 5.65 (1.31) | 8.87 (6.52) | 0.12 (0.12) | 1.13 (0.31) | 0.14 (0.07) | 0.58 (0.03) | 0.07 (0.12) |
| NO3 (mg/kg)*** | 7.48 (3.69) | 19.70 (2.53) | 68.50 (53.01) | 3.15 (0.53) | 1.58 (0.52) | 3.89 (1.39) | 5.39 (4.92) | 2.11 (1.41) | 2.66 (0.73) | 1.18 (0.19) | 1.16 (0.30) | 1.06 (0.17) |
| NH4 (mg/kg)*** | 5.68 (0.32) | 29.90 (14.87) | 13.73 (3.35) | 6.18 (0.66) | 5.19 (0.62) | 9.52 (0.81) | 13.28 (8.92) | 4.65 (0.53) | 5.81 (0.45) | 4.59 (0.51) | 5.47 (0.46) | 5.26 (0.42) |
| T (℃) | 4.8 (1.76) | 7.4 (0.36) | 6.1 (0.23) | 8.2 (0.29) | 6.8 (0.76) | 3.8 (0.00) | 7.6 (0.32) | 8.0 (0.87) | 5.9 (0.75) | 7.7 (0.52) | 8.0 (0.87) | 5.5 (0.50) |
| pH*** | 6.63 (0.10) | 5.63 (0.36) | 6.55 (0.21) | 7.67 (0.15) | 6.72 (0.28) | 5.60 (0.13) | 6.00 (0.26) | 6.93 (0.24) | 6.82 (0.47) | 7.98 (0.09) | 7.73 (0.07) | 7.84 (0.16) |
| Moisture (%)*** | 16.9 (1.3) | 45.3 (19.2) | 21.8 (9.3) | 17.0 (0.9) | 15.3 (2.9) | 21.1 (4.1) | 35.0 (18.2) | 13.4 (2.4) | 17.1 (1.5) | 13.1 (1.5) | 11.5 (0.7) | 19.6 (2.8) |
| Altitude (m) | 11 | 34 | 22 | 42 | 50 | 42 | 47 | 42 | 37 | 32 | 43 | 56 |
| DAC (%)** | 0.21 | 0.11 | 0.38 | 0.20 | 0.02 | 0.14 | 0.50 | 0.10 | 0.31 | 0.02 | 0.02 | 0.03 |
| MS | 4 | 3 | 1 | 2 | 2 | 2 | 2 | 1 | 2 | 0 | 2 | 2 |
| LS | 3 | 8 | 4 | 4 | 6 | 4 | 4 | 2 | 2 | 3 | 5 | 4 |
| VC (%)*** | 75 | 100 | 70 | 30 | 15 | 100 | 95 | 15 | 85 | 8 | 25 | 8 |

**Table S1** Soil elemental compositions and environmental attributes investigated in this study. Abbreviations: T, temperature; DAC, hairgrass (Deschampsia antarctica) coverage; MS, moss species number; and LS, lichen species number. The statistical significance between Group 1 (Q2, Q3, Q6, Q7) and Group 2 (Q1, Q4, Q5, Q9, Q10, Q11, Q12, Q13) was assessed by the non-parametric Wilcoxon test. Significant differences (*P* < 0.05) are indicated in bold. ****P* < 0.001, ***P* < 0.01, **P* < 0.05
