# Supplementary material for: Small-Scale Soil Microbial Community Heterogeneity Linked to Landform Historical Events on King George Island, Maritime Antarctica
Source: Front Microbiol. 2018 Dec 10;9:3065. doi: 10.3389/fmicb.2018.03065 (PMC6296293; doi:10.3389/fmicb.2018.03065)
Supplement: Supplementary file 10 [file Table_3.DOCX]

|  | **Bacteria** | **Archaea** | **Fungi** |
| --- | --- | --- | --- |
| Soil element | *r* value | *r* value | *r* value |
| Al | **0.19**** | **0.21*** | 0.01 |
| Ca | **0.33***** | **0.41***** | 0.01 |
| Cl | **0.48***** | **0.55***** | 0.23 |
| Cu | **0.17***** | 0.10 | 0.16 |
| Fe | **0.40***** | **0.46***** | 0.12 |
| K | **0.18**** | **0.16*** | 0.03 |
| Mg | **0.41***** | **0.37***** | 0.13 |
| Mn | **0.35***** | **0.23**** | 0.09 |
| Na | **0.32***** | **0.21*** | 0.16 |
| P | **0.22**** | **0.36***** | 0.22 |
| S | **0.37***** | **0.50***** | 0.19 |
| Si | **0.36***** | **0.48***** | 0.02 |
| Sr | **0.26**** | **0.34***** | **0.48**** |
| Ti | **0.26***** | **0.29***** | **0.31**** |
| V | 0.013 | 0.07 | 0.021 |
| Zn | **0.19**** | **0.22*** | 0.10 |
| Zr | **0.13*** | 0.00 | 0.14 |
| Ni | 0.08 | -0.12 | -0.01 |
| Br | **0.35***** | **0.57***** | 0.18 |
| Cr | -0.01 | 0.08 | 0.02 |
| Environmental attribute | *r* value | *r* value | *r* value |
| TOC | **0.28***** | **0.443***** | 0.22 |
| NO3 | 0.09 | **0.22*** | -0.01 |
| NH4 | **0.19**** | **0.34***** | 0.08 |
| T | **0.12*** | 0.07 | 0.06 |
| pH | **0.41***** | **0.43***** | **0.54***** |
| Moisture | **0.18**** | **0.31***** | 0.11 |
| Altitude | **0.16*** | -0.06 | **0.32*** |
| DAC | **0.19**** | **0.30**** | 0.02 |
| MS | -0.032 | -0.15 | **0.52***** |
| LS | -0.01 | 0.07 | **0.49***** |
| VC | **0.39***** | **0.35***** | 0.17 |

**Table S3** Mantel test of microbial Operational Taxonomic Unit (OTU) data with soil elemental composition and environmental attributes. Abbreviations: T, temperature; TOC, total organic carbon; MS, moss species amount; LS, lichen species amount; DAC, hairgrass (Deschampsia antarctica) coverage; and VC, total vegetation coverage. Significant differences (*P* < 0.05) are indicated in bold. ****P* < 0.001, ***P* < 0.01, **P* < 0.05.
