# Supplementary material for: Small-Scale Soil Microbial Community Heterogeneity Linked to Landform Historical Events on King George Island, Maritime Antarctica
Source: Front Microbiol. 2018 Dec 10;9:3065. doi: 10.3389/fmicb.2018.03065 (PMC6296293; doi:10.3389/fmicb.2018.03065)
Supplement: Supplementary file 13 [file Data_Sheet_1.pdf]

Figure S1 Pairwise correlative comparisons between soil elements of total samples investigated in this study. Pearson's correlation coefficients are shown on the left lower.

Figure S2 Community composition of (a) bacterial phyla, (b) archaeal classes, and (c) fungal phyla in the 12 quadrats.

Figure S3 Biomass determined by PLFA analyses of each soil quadrat. The total amounts of phospholipid fatty acids (PLFA) were quantified using C19:0 as an internal standard. The *P*-value between Group 1 and Group 2 was calculated by the Wilcoxon test.

Figure S4 Canonical correspondence analysis (CCA) of (a) phospholipid fatty acids (PLFA) data and elemental compositions; and (b) PLFA data and environmental attributes (biomass added as a factor). The percentages of variation explained by each axis and total factors are shown. *P*-values were calculated by the Monte Carlo permutation test (999 permutation).

Figure S5 Significant test of microbial categories marked by PLFA between Group 1 and Group 2.

Figure S6 Neighbour-joining phylogenetic tree made of Operational Taxonomic Unit

(OTU) sequences affiliated to Flavobacteriaceae in this study and an additional 16S rDNA sequence of representative genus Flavobacteriaceae extracted from Genbank. Of 83 OTUs, 7 (otu10028, otu1768, otu32612, otu33716, otu48337, otu69624, otu71597) were only found in Group 1; they have been clustered in the marine clade according to the evolution analysis of the family Flavobacteriaceae by Bowman (2006).

Figure S7 Locations and landscapes of the 12 permanent quadrat plots: Q1, Holatio Cove; Q2, Ardley Island; Q3, Collins Harbour; Q4, Shengwu Cove; Q5, Dajiao Lake; Q6, Great Wall Station; Q7, Nebles Points; Q9, Norma Cove; Q10, Huashan Peninsula; Q11, Haiyan Cove; Q12, Xingfu Cove; and Q13, Longmen Zui.

Table S1 Soil elemental compositions and environmental attributes investigated in this study. Abbreviations: T, temperature; DAC, hairgrass (Deschampsia antarctica) coverage; MS, moss species number; and LS, lichen species number. The statistical significance between Group 1 (Q2, Q3, Q6, Q7) and Group 2 (Q1, Q4, Q5, Q9, Q10, Q11, Q12, Q13) was assessed by the non-parametric Wilcoxon test. Significant differences ($P < 0.05$) are indicated in bold. ***$P < 0.001$, **$P < 0.01$, *$P < 0.05$

Table S2 Microbial Operational Taxonomic Unit (OTU) diversities estimated by the Shannon, Chao1, ACE, and Simpson indices.

Table S3 Mantel test of microbial Operational Taxonomic Unit (OTU) data with soil elemental composition and environmental attributes. Abbreviations: T, temperature; TOC, total organic carbon; MS, moss species amount; LS, lichen species amount; DAC, hairgrass (Deschampsia antarctica) coverage; and VC, total vegetation coverage. Significant differences ($P < 0.05$) are indicated in bold. ***$P < 0.001$, **$P < 0.01$, *$P < 0.05$.

Table S4 Random forest analysis of bacterial, archaeal, and fungal Operational Taxonomic Unit (OTU) data.

Table S5 Pairwise euclidean distances computed between samples with the normalized data of elemental compositions and environmental attributes

Appendix S1. Supplementary Methods
