# Supplementary material for: Small-Scale Soil Microbial Community Heterogeneity Linked to Landform Historical Events on King George Island, Maritime Antarctica
Source: Front Microbiol. 2018 Dec 10;9:3065. doi: 10.3389/fmicb.2018.03065 (PMC6296293; doi:10.3389/fmicb.2018.03065)
Supplement: Supplementary file 14 [file Data_Sheet_2.pdf]

**Supplementary Methods**

The bacterial hypervariable V4 region of the 16S rRNA genes was amplified using primers 515F (5'-GTGCCAGCMGCCGCGGTAA-3') with a 7-nucleotide barcode and 907R (5'-CCGTCAATTCMTTTRAGTTT-3') (Stubner, 2002). Amplifications of the bacterial 16S rRNA genes were performed, consisting of an initial denaturation at 98 °C for 30 s, followed by 25 cycles of denaturation at 98 °C for 15 s, annealing at 50 °C for 30 s, extension at 72 °C for 30 s, and a final extension at 72 °C for 5 min. The archaeal V5-6 region of 16S rRNA genes was amplified using primers 524F-10-extF (5'-TGYCAGCCGCCGCGGTAA-3') with a 7-nucleotide barcode and Arch958-modR (5'-YCCGGCGTTGAVTCCAATT-3') (Pires et al., 2012). Amplifications of the archaeal 16S rRNA genes were performed, consisting of an initial denaturation at 95 °C for 30 s, followed by 25 cycles of denaturation at 95 °C for 15 s, annealing at 55 °C for 30 s, extension at 72 °C for 30 s, and a final extension at 72 °C for 5 min. The fungal rDNA ITS1-5.8S-ITS2 region was amplified using primers ITS5 (5'-GGAAGTAAAAGTCGTAACAAGG-3') with a 7-nucleotide barcode and ITS4 (5'-TCCTCCGCTTATTGATATGC-3') (Baldwin, 1992). Amplifications of the fungal ITS regions were performed, consisting of an initial denaturation at 95 °C for 30 s, followed by 25 cycles of denaturation at 95 °C for 15 s, annealing at 50 °C for 30 s, extension at 72 °C for 30 s, and a final extension at 72 °C for 5 min. The PCR 25 µl reaction mixture contained 0.25 µl Q5 high-fidelity DNA polymerase (NEB), 5 µl reaction buffer, 5 µl high GC buffer, 0.5 µl of 10 mM dNTP, 1 µl template DNA, 1 µl of each primer (10 µM), and 11.25 µl $ddH_2O$.

PCR products were purified using an AxyPreDNA Gel Extraction Kit (Axygen Biosciences, Corning, NY, USA) according to the manufacturer’s instructions. The purified PCR amplicons from each sample were then mixed after quantification using a Quant-iT PicoGreen dsDNA Assay Kit (Invitrogen) in the Microplate reader (Bio Tek, FLx800). Sequencing was performed on the Illumina Miseq Platform.

High-quality clean reads were achieved by removing short reads (<150 bp), long homopolymers (> 8 bp), and reads with ambiguous bases using QIIME software v. 1.8.0 (Boulder, CO, USA). Chimera sequence checking was performed and eliminated by USEARCH v. 5.2.236 (http://www.drive5.com/usearch/). These reads were clustered into OTUs based on 97% sequence similarity using UCLUST, and the most abundant sequence was selected as representative of each OTU. The OTUs with sequence numbers lower than 0.001% of total sequences were removed (Bokulich et al., 2013). The resulting OTU sequences were then aligned with the Greengenes database release 13.8 for bacterial and archaeal 16S rRNA sequences (DeSantis et al., 2006) and the UNITE database release 5.0 for fungal ITS region sequences (Koljalg et al., 2013).

**References:**


Baldwin, B.G. (1992). Phylogenetic Utility of the Internal Transcribed Spacers of Nuclear Ribosomal DNA in Plants: An Example from the Compositae. *Mol. Phylogenet. Evol.* 1, 3-16. doi: 10.1016/1055-7903(92)90030-K.

Bokulich, N.A., Subramanian, S., Faith, J.J., Gevers, D., Gordon, J.I., Knight, R., Mills, D.A., and Caporaso, J.G. (2013). Quality-filtering vastly improves diversity estimates from Illumina amplicon sequencing. *Nat. Methods* 10, 57-U11. doi: 10.1038/NMETH.2276.

DeSantis, T.Z., Hugenholtz, P., Larsen, N., Rojas, M., Brodie, E.L., Keller, K., Huber, T., Dalevi, D., Hu, P., and Andersen, G.L. (2006). Greengenes, a chimera-checked 16S rRNA gene database and workbench compatible with ARB. *Appl. Environ. Microb.* 72, 5069-5072. doi: 10.1128/AEM.03006-05.

Koljalg, U., Nilsson, R.H., Abarenkov, K., Tedersoo, L., Taylor, A.F.S., Bahram, M., Bates, S.T., Bruns, T.D., Bengtsson-Palme, J., Callaghan, T.M., Douglas, B., Drenkhan, T., Eberhardt, U., Duenas, M., Grebenc, T., Griffith, G.W., Hartmann, M., Kirk, P.M., Kohout, P., Larsson, E., Lindahl, B.D., Luecking, R., Martin, M.P., Matheny, P.B., Nguyen, N.H., Niskanen, T., Oja, J., Peay, K.G., Peintner, U., Peterson, M., Poldmaa, K., Saag, L., Saar, I., Schuessler, A., Scott, J.A., Senes, C., Smith, M.E., Suija, A., Taylor, D.L., Telleria, M.T., Weiss, M., and Larsson, K. (2013). Towards a unified paradigm for sequence-based identification of fungi. *Mol. Ecol.* 22, 5271-5277. doi: 10.1111/mec.12481.

Pires, A.C.C., Cleary, D.F.R., Almeida, A., Cunha, A., Dealtry, S., Mendonca-Hagler,

L.C.S., Smalla, K., and Gomes, N.C.M. (2012). Denaturing Gradient Gel Electrophoresis and Barcoded Pyrosequencing Reveal Unprecedented Archaeal Diversity in Mangrove Sediment and Rhizosphere Samples. *Appl. Environ. Microb.* 78, 5520-5528. doi: 10.1128/AEM.00386-12.

Stubner, S. (2002). Enumeration of 16S rDNA of Desulfotomaculum lineage 1 in rice field soil by real-time PCR with SybrGreen (TM) detection. *J. Microbiol. Meth.* 50, 155-164. doi: 10.1016/S0167-7012(02)00024-6.
